# A Potential Approach of Mesenchymal Stem Cells Combined Silybin for Synergistic Treatment in Rheumatoid Arthritis via ICOS/ICOSL

**DOI:** 10.1002/mco2.70450

**Published:** 2025-10-28

**Authors:** Yu Chun Wang, Shuai Ding, Ya Feng Wang, Han Xie, Shan Shan Liu, Hong Wei Chen, Dan Wu, Ying Xie, Xin Wen, Yi Zhun Zhu, Ling Yun Sun

**Affiliations:** ^1^ The Key Laboratory of Biochemistry and Molecular Pharmacology Chongqing Medical University Chongqing China; ^2^ School of Pharmacy Faculty of Medicine Macau University of Science and Technology Taipa Macau SAR China; ^3^ Department of Rheumatology and Immunology Nanjing Drum Tower Hospital Nanjing Jiangsu China; ^4^ The Second Affiliated Hospital of Guangzhou University of Chinese Medicine Guangzhou Guangdong China

**Keywords:** ICOS/ICOSL, rheumatoid arthritis, silybin, UC‐MSCs

## Abstract

Achieving clinical remission in rheumatoid arthritis (RA) remains a significant challenge in current therapeutic strategies. While transplantation of human umbilical cord‐derived mesenchymal stem cells (UC‐MSCs) has shown promising outcomes, therapeutic responses vary considerably among patients. In this study, we characterized the immune profiles of nonresponders and identified elevated expression of inducible costimulator (ICOS) in peripheral immune cells as a critical barrier to effective treatment. This upregulation, combined with the presence of ICOS ligand (ICOSL) on UC‐MSCs, activated T cells to secrete inflammatory cytokines through the Phosphatidylinositol 3‐kinase / Protein kinase B / Mammalian target of rapamycin (PI3K/AKT/mTOR) pathway. To overcome this limitation, we identified silybin as a potential adjunctive agent. Further investigations demonstrated that silybin acts as a competitive binding inhibitor, effectively targeting the PI3K/AKT/mTOR pathway and reducing downstream cytokine release. The combined application of silybin and UC‐MSCs significantly enhanced immunoregulatory effects, as validated through in vitro analyses with patient‐derived samples and in vivo experiments using a collagen‐induced arthritis mouse model. This study highlights a novel, personalized therapeutic approach for RA, offering insights into improving clinical outcomes through targeted interventions.

## Graphical abstract

In our study, UC‐MSCs showed a poor immunoregulatory effect for RA patients with upregulated inducible costimulator (ICOS) in PBMCs. And we identified the ICOS ligand (ICOSL) of UC‐MSCs combined with ICOS of RA patients, activating Phosphatidylinositol 3‐kinase / Protein kinase B / Mammalian target of rapamycin (PI3K/AKT/mTOR). The CM5 chip was installed according to the standard operating procedures of the Biacore 8k instrument prior to ligand immobilization. pathway and triggering the subsequent inflammatory cascade reaction. Besides, silybin (SB) could directly bind to ICOS in PBMCs of RA patients competing with UC‐MSCs. Finally, we found a combination of UC‐MSCs and SB alleviated RA better in the CIA model, providing a new approach for treating RA.

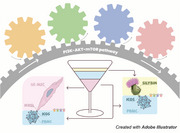

## 1 Introduction

Rheumatoid arthritis (RA) is a chronic autoimmune disease primarily afflicting the synovial membranes, cartilage, and bones of the hands, feet, and knees [1, 2]. The principal pathogenic mechanisms involve systemic and synovial inflammation stemming from imbalanced immune homeostasis [3]. Current clinical management of RA encompasses diverse interventions, including disease‐modifying antirheumatic drugs, nonsteroidal anti‐inflammatory drugs (NSAIDs), glucocorticoids, and traditional Chinese medicine. Nonetheless, the curative outcomes of these treatments remain unsatisfactory.

For the past decade, human umbilical cord‐derived mesenchymal stem cells (UC‐MSCs), as multipotent stem cells, have emerged as a promising avenue for treating autoimmune and inflammatory diseases, demonstrating optimal safety and efficacy in clinical settings [4, 5, 6]. While UC‐MSCs possess a remarkable ability to modulate various immune cell types, there have also been reports documenting instances of treatment inefficacy. In a multicenter clinical follow‐up study spanning 12 months, 40% of patients with SLE showed no clinical response after MSC infusion [7]. Within a cohort subjected to UC‐MSCs transplantation, 28 RA patients exhibited a notable amelioration of clinical symptoms, alongside reduced disease activity and drug dosages over the 12‐week post‐MSCT, whereas 24 RA patients exhibited no discernible improvement [8]. Among the numerous factors influencing MSCT clinical outcomes, the heterogeneity of UC‐MSC subpopulations has emerged as a critical determinant of treatment efficacy, yet remains insufficiently understood. In our previous research, scRNA‐seq analysis identified distinct clusters‐primed, intermediate, and stem‐like states‐each exhibiting varying degrees of immunosuppressive activity [9]. The heterogeneity in UC‐MSC subpopulations contributes to variability in treatment outcomes; however, patient‐specific factors also play a crucial role. For instance, our previous study revealed that IFN‐γ levels in patients can significantly influence the therapeutic efficacy of UC‐MSCs [10]. Furthermore, the diverse immune disorder characteristics in patients contribute to the disease's complexity and treatment failure [11, 12, 13]. Our previous study found that IFN‐r levels in patients can affect UC‐MSC efficacy [10]. For instance, molecular epidemiological studies have highlighted the close association between CD28/cytotoxic T‐lymphocyte‐associated protein 4/inducible costimulator (ICOS) polymorphisms and susceptibility to RA in patients [11]. Mechanistically, ICOS signaling plays a crucial role in both the initiation and persistence of collagen‐induced arthritis (CIA) in mouse models. Consequently, there is a growing emphasis on elucidating the distinct immune characteristics of individuals with suboptimal treatment responses.

RA presents a complex therapeutic scenario due to a convergence of interrelated factors, including heterogeneity of disease, elusive etiology, and tissue specificity [14, 15]. In light of these challenges, the utilization of different approaches in combination holds promise for maximizing therapeutic effects. In a murine model of CIA, the concomitant administration of bone marrow‐derived mesenchymal stem cells (BM‐MSCs) and interleukin‐4 (IL‐4) demonstrated notable enhancements in ameliorating RA symptoms [16]. However, in clinical practice, the implementation of combination therapy presents several challenges, including dosage optimization, safety concerns, potential drug interactions, and side effects. Most critically, a lack of understanding of the underlying mechanisms complicates efforts to refine and optimize therapeutic strategies.

In optimizing UC‐MSC therapy for RA, our study identified elevated ICOS expression in peripheral immune cells as a key challenge in nonresponders. Through an in vitro coculture system and cytokine profiling, we confirmed that ICOS engagement with its ligand (ICOSL) on UC‐MSCs activates T cells, driving inflammatory cytokine release via the PI3K/AKT pathway. Using both engineered cell lines and patient‐derived samples, we further validated this mechanism. To mitigate these effects, we employed computational modeling to screen for competitive ICOS/ICOSL inhibitors and identified silybin (SB), a compound with ICOS‐binding properties, as a potential enhancer of UC‐MSC function. The main component of SB, a flavonoid extracted from the fruit of Juli plant flying thistle (Silybum marianum), has obvious effects of protecting and stabilizing liver cell membrane, can improve liver function, produce enzyme lowering effect, is not easy to occur enzyme rebound, and has been widely used in clinical practice [17, 18]. Our findings revealed that SB significantly augmented the immunoregulatory effects of UC‐MSCs, both in vitro with RA patient‐derived peripheral immune cells and in vivo using the CIA model. Collectively, our results highlight the potential of combining SB with UC‐MSCs as a promising therapeutic strategy for RA.

## 2 Results

### 2.1 ICOS/ICOSL Expression and Cytokine Dynamics Define UC‐MSC Treatment Outcomes in RA

**FIGURE 1:**
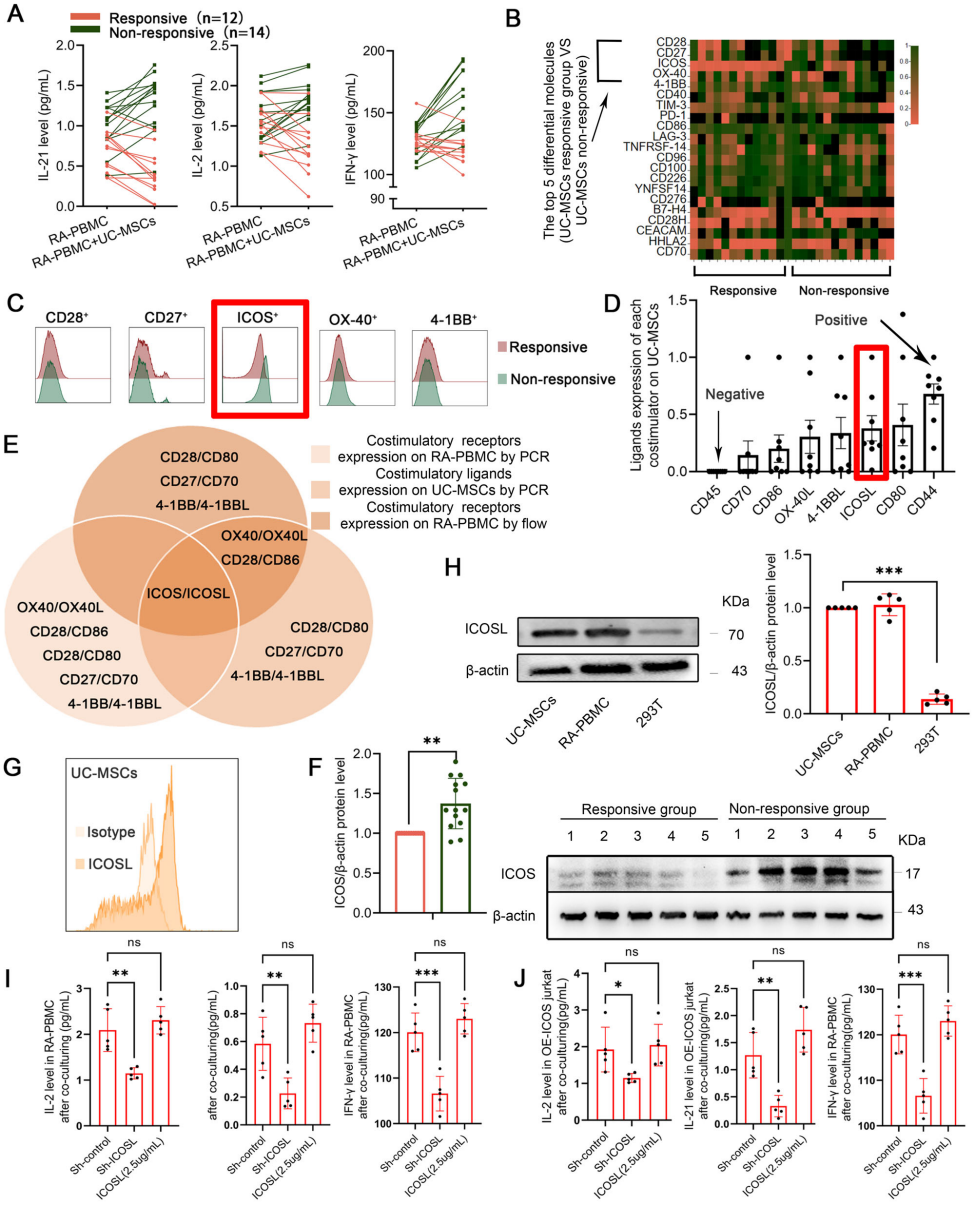
ICOS/ICOSL expression and cytokine dynamics define UC‐MSC treatment outcomes in RA. (A) Levels of IFN‐γ, IL‐2, and IL‐21 in RA‐PBMC culture supernatants before and after coculture with UC‐MSCs. Patients were categorized as the responsive group (*n* = 12) if cytokine levels decreased poststimulation or the nonresponsive group (*n* = 14) if levels remained unchanged or increased (total *n* = 26). (B) Heatmap of differentially expressed genes related to T cell activation in PBMCs from UC‐MSC responders (*n* = 12) and nonresponders (*n* = 14). The top five genes shown exhibit the most significant differences between the two groups. (C) Flow cytometry analysis of the top five differentially expressed genes in PBMCs from UC‐MSC responders (*n* = 12) and nonresponders (*n* = 14), with ICOS showing the most significant difference. (D) Expression of ligands on UC‐MSCs corresponding to the top five differentially expressed genes. CD45 was used as a negative control (no expression), while CD44 served as a positive control (high expression). (E) ICOS/ICOSL emerged as the most distinct costimulatory pair differentiating UC‐MSC responders from nonresponders. This selection was based on the intersection of differentially expressed genes in patient PBMCs and their corresponding ligands on UC‐MSCs, identified through heatmap, flow cytometry, and PCR analysis. (F) Western blot analysis of ICOS expression in PBMCs from UC‐MSC responders and nonresponders (*n* = 12 and 14). Data represent one of two independent experiments, showing elevated ICOS expression in the nonresponsive group. (G) Expression of ICOSL on UC‐MSCs FACS comparing with isotype. (H) Western blot of ICOSL on UC‐MSCs comparing with human‐PBMC and 293T cell line. (I) The levels of IFN‐γ, IL‐2, and IL‐21 in cell culture supernatant in RA‐PBMC after coculturing with sh‐control or sh‐ICOSL UC‐MSCs or ICOSL(2.5 µg/mL) (*n* = 5). (J) The levels of IFN‐γ, IL‐2, and IL‐21 in cell culture supernatant of OE‐ICOS Jurkat T cells after coculturing with sh‐control or sh‐ICOSL UC‐MSCs or ICOSL(2.5 µg/mL) (*n* = 5). Data are represented as mean ± SEM.

To discern the characteristics of RA patients who did not show improvement post‐UC‐MSCs transplantation, we established an in vitro coculture system. The system classifies RA patients based on the cytokines secreted into the culture supernatants after 24 h, specifically IL‐21, IL‐2, and IFN‐γ. These cytokines are intricately associated with RA progression and typically show marked reduction corresponding with symptom relief in clinical scenarios [19, 20]. Based on these criteria, out of 26 individuals, we identified 12 patients as UC‐MSC responders, evidenced by a significant reduction in the levels of IFN‐γ, IL‐21, and IL‐2. Conversely, the remaining 14 individuals exhibited an increase in these cytokine levels as UC‐MSC nonresponders (Figure 1A). Following the categorization of patients based on cytokine secretion, we quantified the expression of costimulatory factors, pivotal molecules that augment immune cell activation, and might serve as upstream modulators driving the overproduction of cytokines like IFN‐γ, IL‐21, and IL‐2 [21]. Utilizing qPCR to detect CD28, CD27, ICOS, OX‐40, 4‐1BB, CD40, TIM‐3, PD‐1, CD86, LAG‐3, TNFRSF‐14, CD96, CD100, CD226, YNFSF14, CD276, B7‐H4, CD28H, CEACAM, HHLA2, and CD70 [22, 23, 24, 25, 26, 27], we observed that the expression levels of costimulatory molecules CD28, CD27, ICOS, OX‐40, and 4‐1BB varied notably between the two groups (Figure 1B). Specifically, their expression positively correlated with the levels of released cytokines [20, 28, 29]. Concurrently, we utilized flow cytometry to validate the expression levels of CD28, CD27, ICOS, OX‐40, and 4‐1BB, and the findings aligned with those from the qPCR analysis (Figure 1C), suggesting that these molecules may potentially be associated with the effectiveness of UC‐MSCs treatment.

Given that the activity of costimulatory molecules is dependent on ligand binding, we subsequently evaluated the expression of costimulatory factor ligands on UC‐MSCs. We focused on CD80/CD86, ICOSL, OX‐40L, CD70, and 4‐1BBL, which correspond to the distinct molecules that we identified as varying between the two groups. From qPCR analysis, both CD80 and ICOSL exhibited relatively higher expression levels on MSCs (Figure 1D). Notably, when identifying intersections between the most distinct costimulatory molecules and their correspondingly highly expressed ligands, ICOSL emerged as the most prominent one (Figure 1E).

We subsequently examined the expression of ICOS via Western blot and observed a notable increase of ICOS in the UC‐MSC nonresponsive group compared with the responsive group, as illustrated in Figure 1F. Additionally, using FACS, we probed ICOSL expression in UC‐MSCs and also compared its levels in UC‐MSCs, PBMCs isolated from patients, and 293T cells (Figure 1G,H). These assessments aligned with previous qPCR findings, showing a relatively higher expression of ICOSL. To further substantiate our hypothesis that ICOS/ICOSL are pivotal determinants of UC‐MSC responsiveness, we generated ICOSL‐knockdown UC‐MSCs and employed the in vitro coculture system, supplemented with ICOSL recombinant protein as a positive control. Cytokine levels of IFN‐γ, IL‐21, and IL‐2 decreased significantly after ICOSL knockdown in UC‐MSCs. Cytokine levels of IFN‐γ, IL‐21, and IL‐2 decreased significantly after ICOSL knockdown in UC‐MSCs (Figure 1I). Additionally, we established an ICOS overexpression cell line to simulate the elevated ICOS expression observed in nonresponsive patients. A similar reduction was observed in ICOSL‐knockdown UC‐MSCs upon coculture completion (Figure 1J). Concordant costimulatory molecules associated with T cell activation on PBMC bound to their ligands on UC‐MSCs playing a negative role in RA.

### 2.2 Activation of the PI3K/AKT/mTOR Pathway in RA Mediated by UC‐MSCs Through ICOS/ICOSL Interaction

**FIGURE 2:**
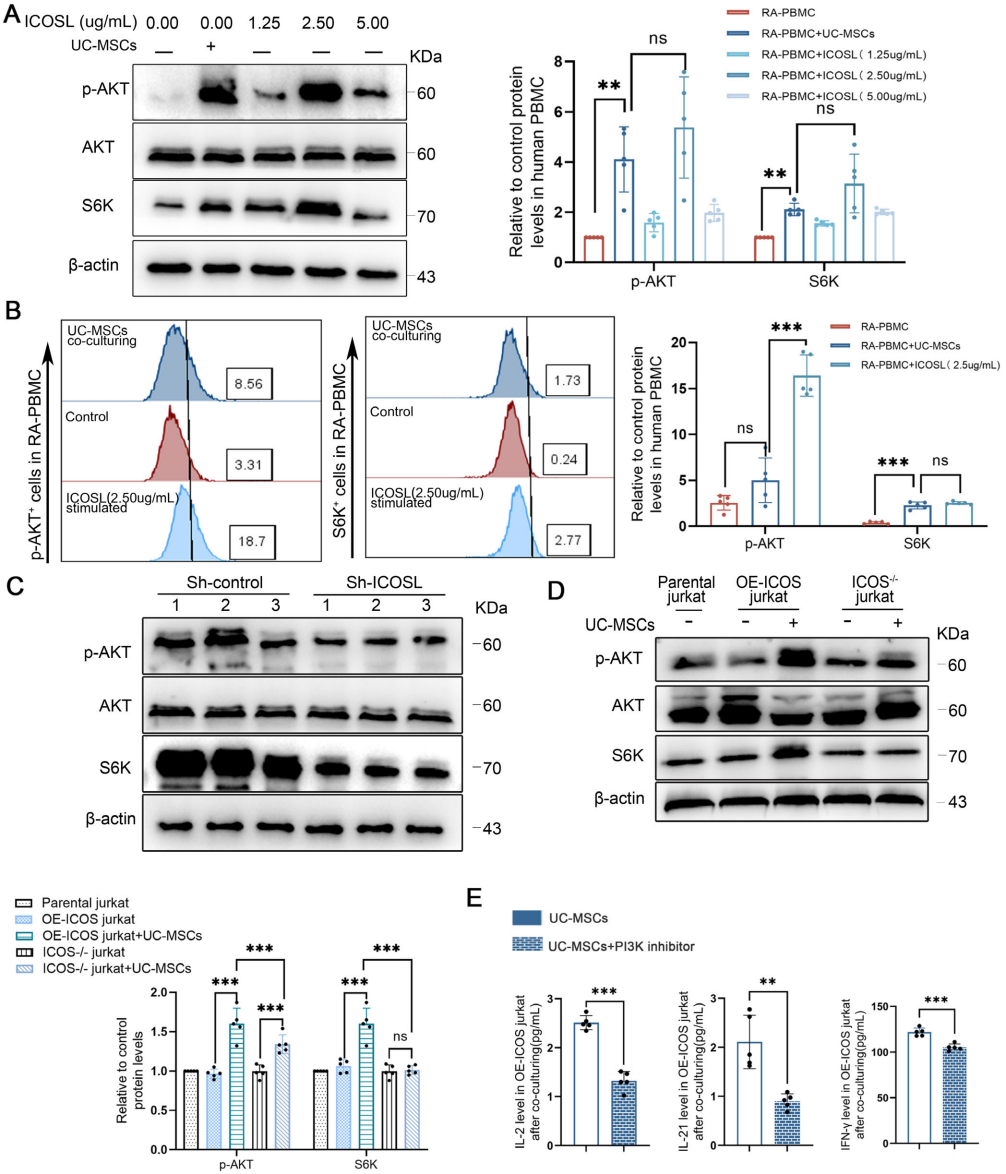
Activation of the PI3K/AKT/mTOR pathway in RA mediated by UC‐MSCs through ICOS/ICOSL interaction. (A) Western blot analysis of p‐AKT, total AKT, and S6K in RA‐PBMCs after coculture with UC‐MSCs or ICOSL (0, 2.5, and 5 µg/mL) (*n* = 5), confirming activation of this pathway by both UC‐MSCs and ICOSL. (B) Expression of p‐AKT and S6k in RA‐PBMC after coculturing with UC‐MSCs or ICOSL (2.5 µg/mL) by FACS (*n* = 5). (C) Western blot analysis of p‐AKT, total AKT, and S6K in RA‐PBMCs after coculture with sh‐control or sh‐ICOSL UC‐MSCs (*n* = 5), demonstrating that ICOSL knockdown in UC‐MSCs abolishes pathway activation. (D) Western blot analysis of p‐AKT, total AKT, and S6K in parental Jurkat, OE‐ICOS Jurkat, and ICOS^−/−^ Jurkat T cells after coculture with UC‐MSCs (*n* = 5), showing that ICOS knockout prevents pathway activation, whereas ICOS overexpression significantly enhances it. (E) The levels of IFN‐γ, IL‐2, and IL‐21 in cell culture supernatant of OE‐ICOS Jurkat T cells after coculturing with UC‐MSCs alone or UC‐MSCs plus PI3K inhibitor (*n* = 5). Data are represented as mean ± SEM.

As a canonical T‐cell activation axis, the ICOS/ICOSL interaction modulates T‐cell survival, proliferation, and functionality primarily through the PI3K/AKT/mTOR signaling pathway. Given the ICOSL expression in UC‐MSCs, we investigated whether UC‐MSCs could stimulate the PI3K/AKT/mTOR pathway in PBMCs post coculture. Our results indicated that UC‐MSCs elevated the protein concentrations of p‐AKT and S6K, surpassing even the effects of the ICOSL recombinant protein (Figure 2A). To rule out the influence of endogenous PI3K/AKT/mTOR molecules in MSCs, we assessed S6K and p‐AKT levels using FACS within the PBMCs of RA patients (Figure 2B). These results aligned with our Western blot data. Furthermore, there was a significant reduction in S6K and p‐AKT levels upon ICOSL knockdown in UC‐MSCs, an effect comparable to that of a PI3K inhibitor (Figure 2C).

To ascertain whether the PI3K/AKT/mTOR pathway is primarily activated via an ICOS‐dependent mechanism, we generated an ICOS‐deficient Jurkat cell line, and then cocultured naive Jurkat cells and ICOS‐related derivatives with UC‐MSCs under identical conditions. Our data revealed that ICOS inactivation significantly reduces p‐AKT and S6K levels (Figure 2D), suggesting its central role in modulating responses to UC‐MSC. Additionally, utilizing IFN‐γ, IL‐21, and IL‐2 as functional readouts and a PI3K inhibitor as a positive control, our analyses underscored that both ICOS and ICOSL are crucial in elucidating the activation of the PI3K/AKT/mTOR pathway and the subsequent overproduction of some cytokines, specifically IFN‐γ, IL‐21, and IL‐2 (Figure 2E).

**FIGURE 3:**
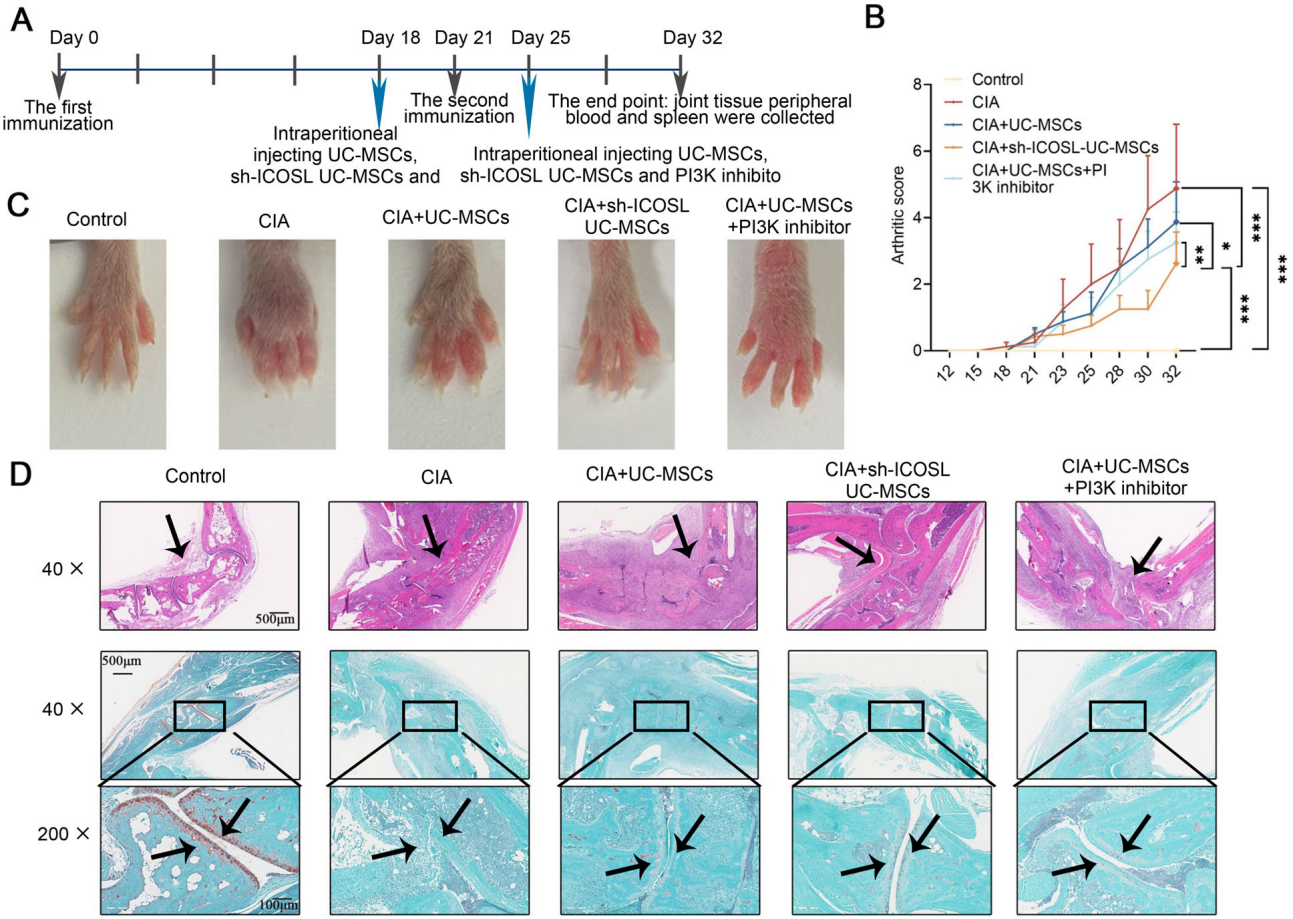
Enhanced protective effect of ICOSL‐deficient UC‐MSCs in CIA mice. (A) Schematic representation of the animal experiments. (B) The arthritis scores for CIA. (C) Representative photographs of hind paws. (D) Histopathological images (magnification ×4) of hind paws. (E) Red solid green staining (magnification ×4, ×10). Data are represented as mean ± SEM (*n* = 8).

To assess the in vivo feasibility and efficacy of the ICOSL on UC‐MSCs and PI3K inhibitor, we established a CIA model in DBA/1 mice using two intradermal injections of type II collagen on days 0 and 21. During the induction phase, mice were treated with either UC‐MSCs, sh‐ICOSL UC‐MSCs, or UC‐MSCs plus PI3K inhibitor (Figure 3A). As expected, sh‐ICOSL UC‐MSCs or UC‐MSCs plus PI3K inhibitor alleviated the arthritic symptoms in CIA mice better than UC‐MSCs alone, as evidenced by reduced arthritic scores, decreased paw swelling, and improved histological features (Figure 3B–E).

### 2.3 SB‐Mediated Disruption of ICOS–ICOSL Interaction Augments UC‐MSCs Therapeutic Efficacy

**FIGURE 4:**
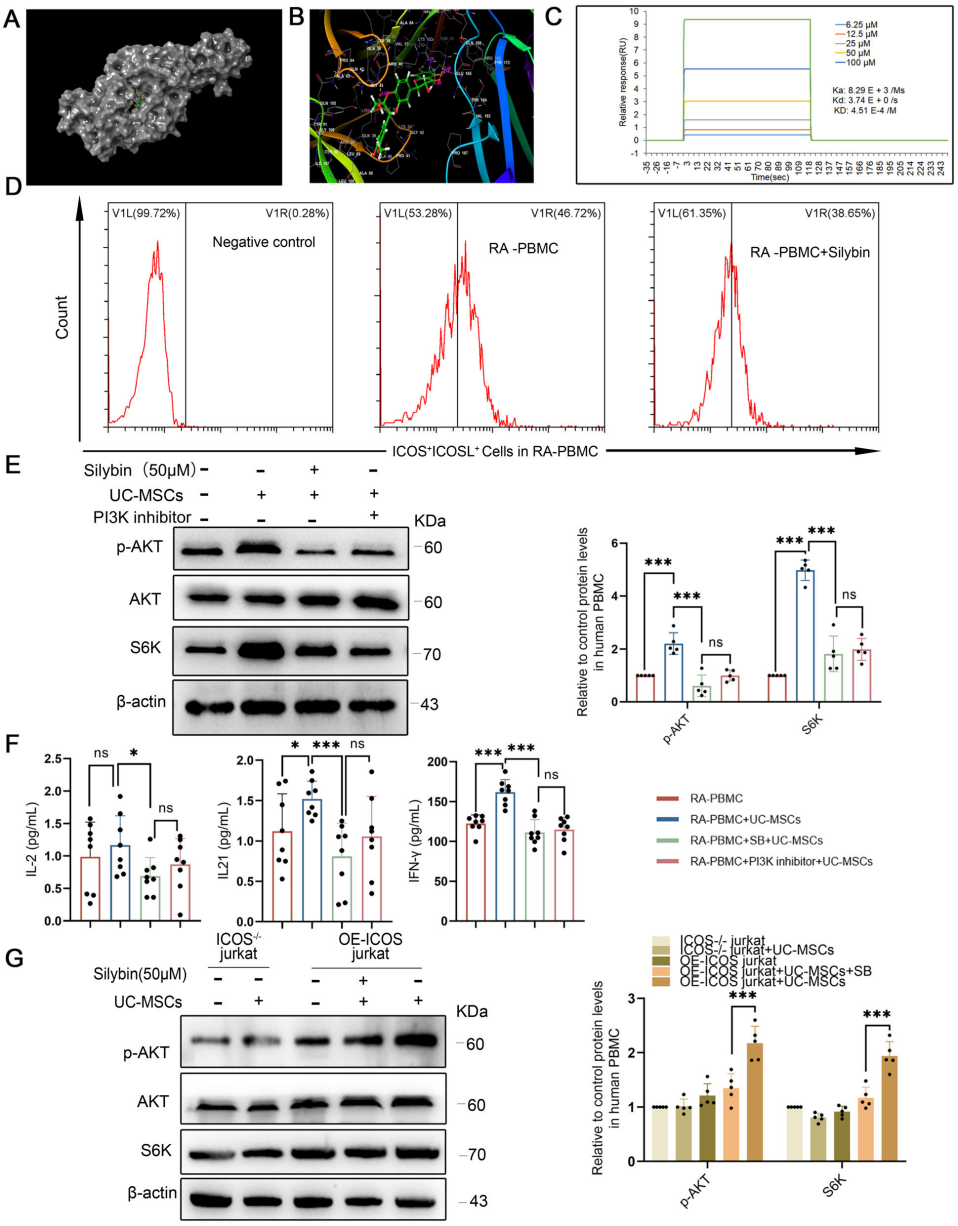
Silybin‐mediated disruption of ICOS–ICOSL interaction augments UC‐MSCs therapeutic efficacy. (A and B) Docking simulation analysis identified two potential silybin binding sites on ICOS. (C) Surface plasmon resonance (SPR) confirmed silybin's direct interaction with ICOS. (D) Flow cytometry analysis showed reduced ICOS–ICOSL binding in PBMCs pretreated with silybin, indicating competitive inhibition. (E) Western blot analysis revealed that silybin significantly inhibited p‐AKT and S6K levels in the UC‐MSC–PBMC coculture system, similar to a PI3K inhibitor. (F) ELISA demonstrated that silybin suppressed IL‐2, IL‐21, and IFN‐γ secretion from RA patient PBMCs. (G) Coculture experiments with Jurkat T cells and ICOS‐deficient derivatives confirmed that silybin's inhibitory effect on the PI3K/AKT/mTOR pathway is ICOS‐dependent. These findings suggest that silybin enhances UC‐MSC therapeutic efficacy by competitively binding to ICOS and mitigating immune activation. Data are represented as mean ± SEM (*n* = 5).

We pinpointed a critical challenge with nonresponders: the inappropriate binding of elevated ICOS on T cells to ICOSL on UC‐MSCs, leading to subsequent unwanted immune activation. To address this, we extensively reviewed both current clinical medications and experimental treatments, aiming to identify a therapy that could counteract this undesired effect. From our screening, SB stood out as a promising candidate, as demonstrated by the docking simulation analysis (Figure 4A,B). SB exhibited potential interactions with ICOS at two binding sites, the finding that was experimentally validated using surface plasmon resonance (SPR) experiments (Figure 4C). To assess whether SB interferes with ICOS–ICOSL interactions, PBMCs from refractory RA patients were divided into two groups, with one pretreated with SB for 24 h. Both groups were then incubated with Fc‐tagged recombinant ICOSL, followed by fluorescent anti‐Fc antibody labeling. Flow cytometry revealed significantly reduced ICOS–ICOSL binding in the SB‐pretreated group, suggesting that SB may competitively inhibit ICOS engagement (Figure 4D).

Subsequently, we explored whether SB could modulate the PI3K/AKT/mTOR pathway via ICOS. Our results revealed that in the presence of SB, the protein levels of p‐AKT and S6K were significantly inhibited in the UC‐MSCs and PBMCs coculture system, mirroring the effects of a PI3K inhibitor (Figure 4E). Concurrently, the secretion of IL‐2, IL‐21, and IFN‐γ from PBMCs in RA patients was markedly abolished (Figure 4F). These findings suggest that SB's effects are similar to those of a PI3K inhibitor, likely due to its direct binding to ICOS.

To further substantiate that SB modulates the PI3K/AKT/mTOR pathway activation through ICOS, we employed Jurkat T cells and ICOS‐deficient derivatives in a coculture with UC‐MSCs and SB. This confirmed that SB inhibits the PI3K/AKT/mTOR pathway in an ICOS‐dependent manner (Figure 4G). In summary, SB attenuates the PI3K/AKT/mTOR pathway and diminishes cytokine release by competitively binding to ICOS on PBMCs of RA patients, offering potential enhancements to UC‐MSCs therapeutic outcomes.

### 2.4 SB Boosted UC‐MSCs’ Immunoregulatory Effect in Vitro for RA Patients

**FIGURE 5:**
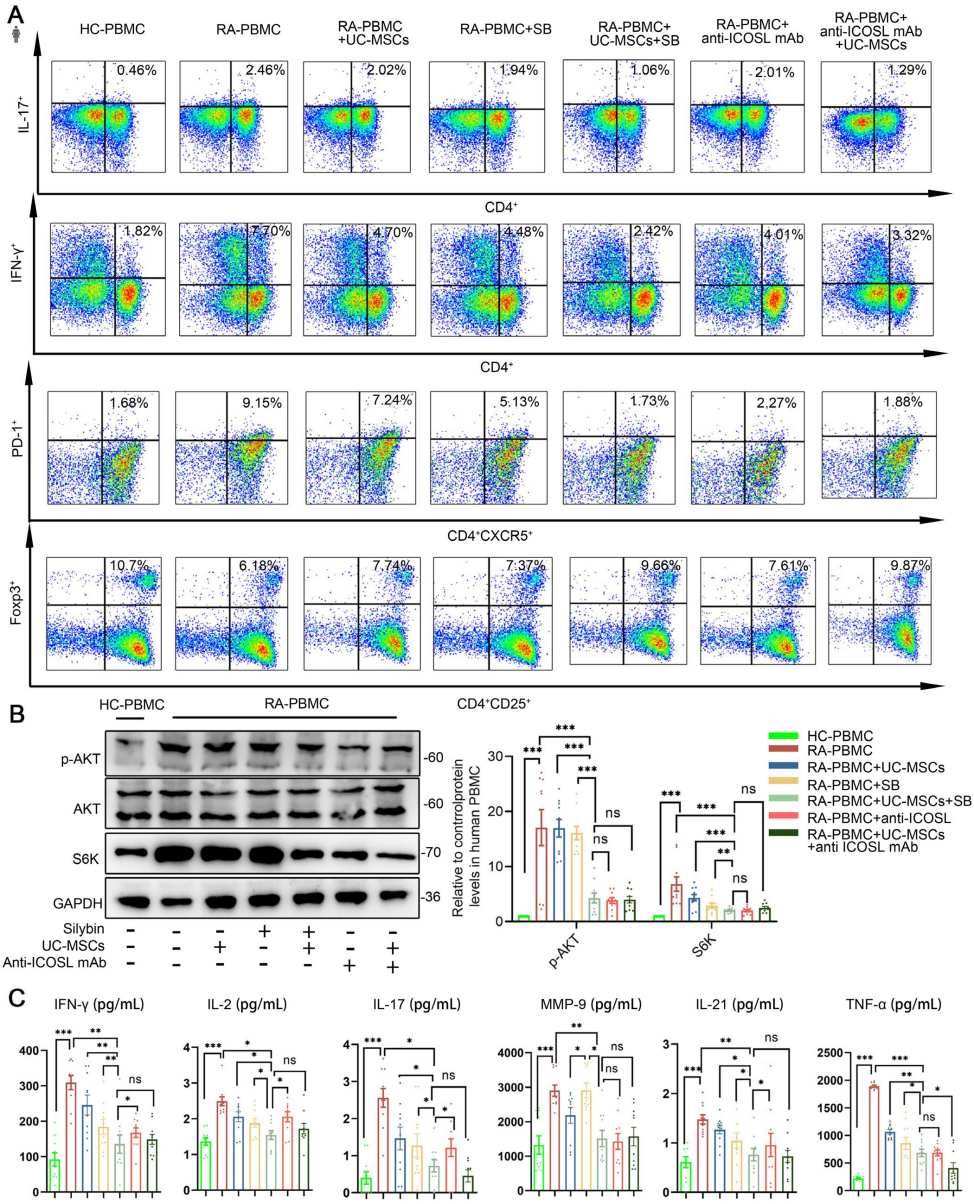
Silybin boosted UC‐MSCs’ immunoregulatory effect in vitro for RA patients. (A) IL‐17A^+^CD4^+^Th17, IFN‐γ^+^CD4^+^Th1, and PD1^+^CXCR5^+^CD4^+^Tfh cells proportion of each group of in healthy control and RA PBMC. (B) Western blot of p‐AKT, total AKT, and S6k in healthy control and RA‐PBMC. (C) Expression of inflammatory factors in each group of cell culture supernatant. Data are represented as mean ± SEM (*n* = 10).

To evaluate the efficacy of the combination therapy, we cocultured PBMCs from RA patients with either UC‐MSCs, SB, or a combination of both. We then measured the functional subsets across these groups by FACS. Our findings indicated that Th17, Th1, and Tfh cell populations were notably elevated in RA patients compared with the healthy control (HC) group. However, these increases were mitigated following treatments, with the combination of UC‐MSCs and SB exhibiting the most pronounced effect (Figure 5A). Subsequently, to ascertain if the suppression of these T cells was associated with the PI3K/AKT/mTOR pathway, we examined the protein levels of p‐AKT and S6K (Figure 5B). The results aligned with our previous observations, indicating that the combination of UC‐MSCs and SB was the most effective in inhibiting the PI3K/AKT/mTOR pathway. Additionally, in the cell culture supernatant, we noted a decrease in the levels of IFN‐γ, IL‐2, and IL‐21 in the combination group compared with the group treated with UC‐MSCs alone (Figure 5C). Blocking ICOS/ICOSL interaction with anti‐ICOSL mAb led to an improved immune cell profile, particularly when combined with UC‐MSC stimulation, resulting in enhanced therapeutic effects. These findings are consistent with the observed pathway activation and cytokine secretion patterns.

### 2.5 A Combination Strategy of UC‐MSCs and SB Alleviated Arthritic Symptoms in the CIA Model via ICOS/ICOSL

**FIGURE 6:**
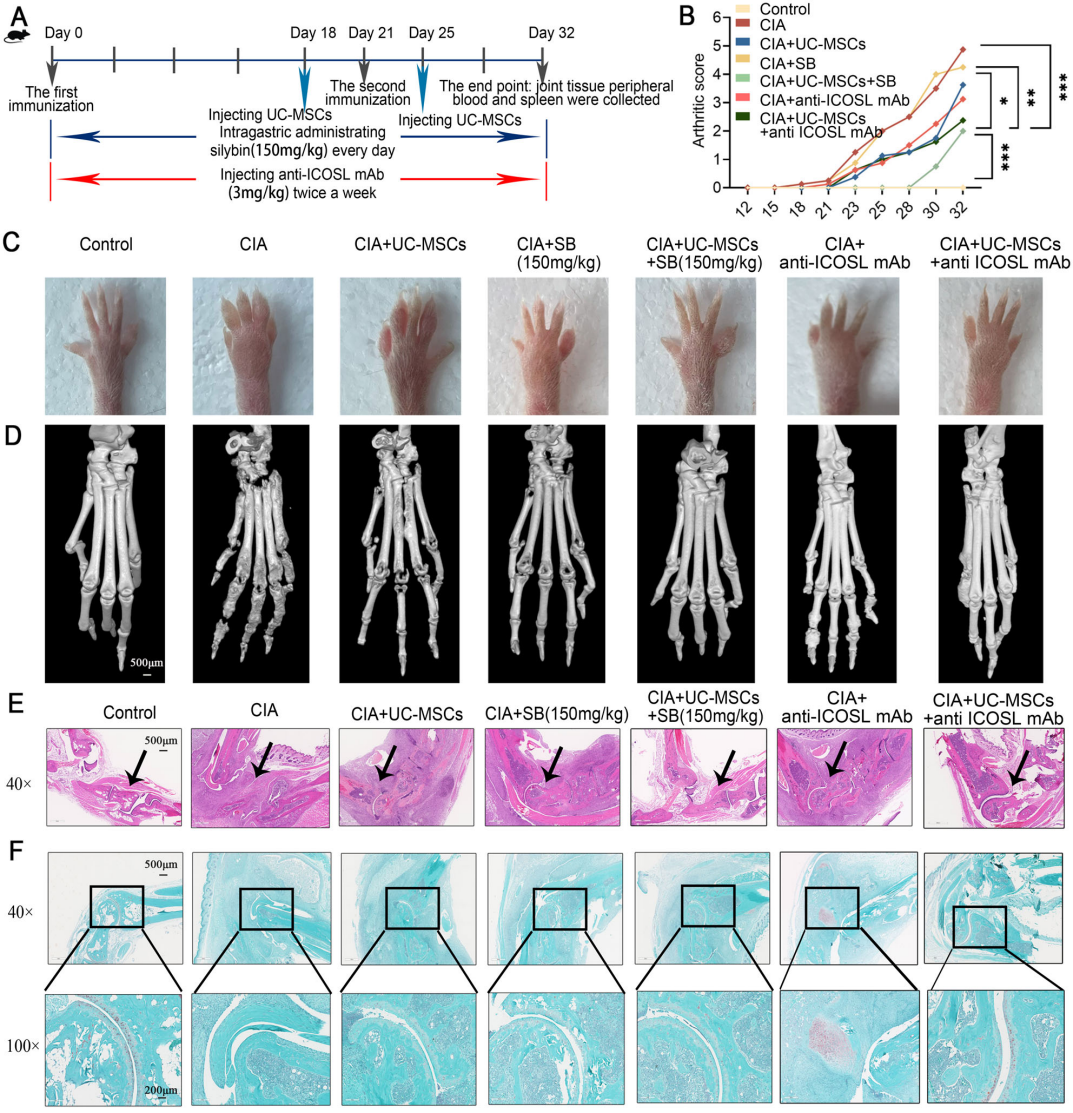
A combination strategy of UC‐MSCs and silybin alleviated arthritic symptoms in the CIA model. (A) Schematic representation of the collagen‐induced arthritis (CIA) model induction and treatment regimen in DBA/1 mice. (B) Arthritic scores over time demonstrated that the combination of UC‐MSCs and silybin (SB) significantly alleviated disease severity and delayed onset. (C) Paw swelling measurements and histological analysis revealed superior therapeutic effects in the combination treatment group compared with single treatments. (D) Micro‐CT analysis of bone destruction, showing increased trabecular bone thickness (Tb.Th) and decreased bone surface area to bone volume ratio (BS/BV) in the combination group, indicative of reduced bone damage. (E and F) Histological scoring further confirmed the enhanced protective effects of coadministration. These results highlight the potential of combining UC‐MSCs with silybin to improve clinical outcomes in RA treatment.

To assess the in vivo feasibility and efficacy of the combined treatment strategy, we established a CIA model in DBA/1 mice using two intradermal injections of type II collagen on days 0 and 21. During the induction phase, mice were treated with either UC‐MSCs, SB, or a combination of both (Figure 6A). As expected, UC‐MSCs alleviated the arthritic symptoms in CIA mice, as evidenced by reduced arthritic scores, decreased paw swelling, and improved histological features (Figure 6B–F). Remarkably, the combined treatment of UC‐MSCs and SB exhibited superior therapeutic effects compared with either treatment alone (Figure 6B–F). Encouragingly, this coadministration also delayed disease onset by a week (Figure 6B). To better evaluate the impact on the most affected organ, we employed micro‐CT to visualize bone destruction in the DBA/1 mouse and assessed bone mass density using two metrics: trabecular bone thickness (Tb.Th) and bone surface area to bone volume ratio (BS/BV). It is worth noting that a lower Tb.Th value indicates significant bone damage, while a higher BS/BV ratio suggests increased bone cavitation and severe damage. Our findings revealed an increase in Tb.Th and a decrease in BS/BV for the combination treatment group relative to the single‐treatment groups (Figures 6D and S1), supporting the combined strategy's potential. ICOSL blockade in mice provided protective effects and further amplified the therapeutic benefits of UC‐MSCs.

**FIGURE 7:**
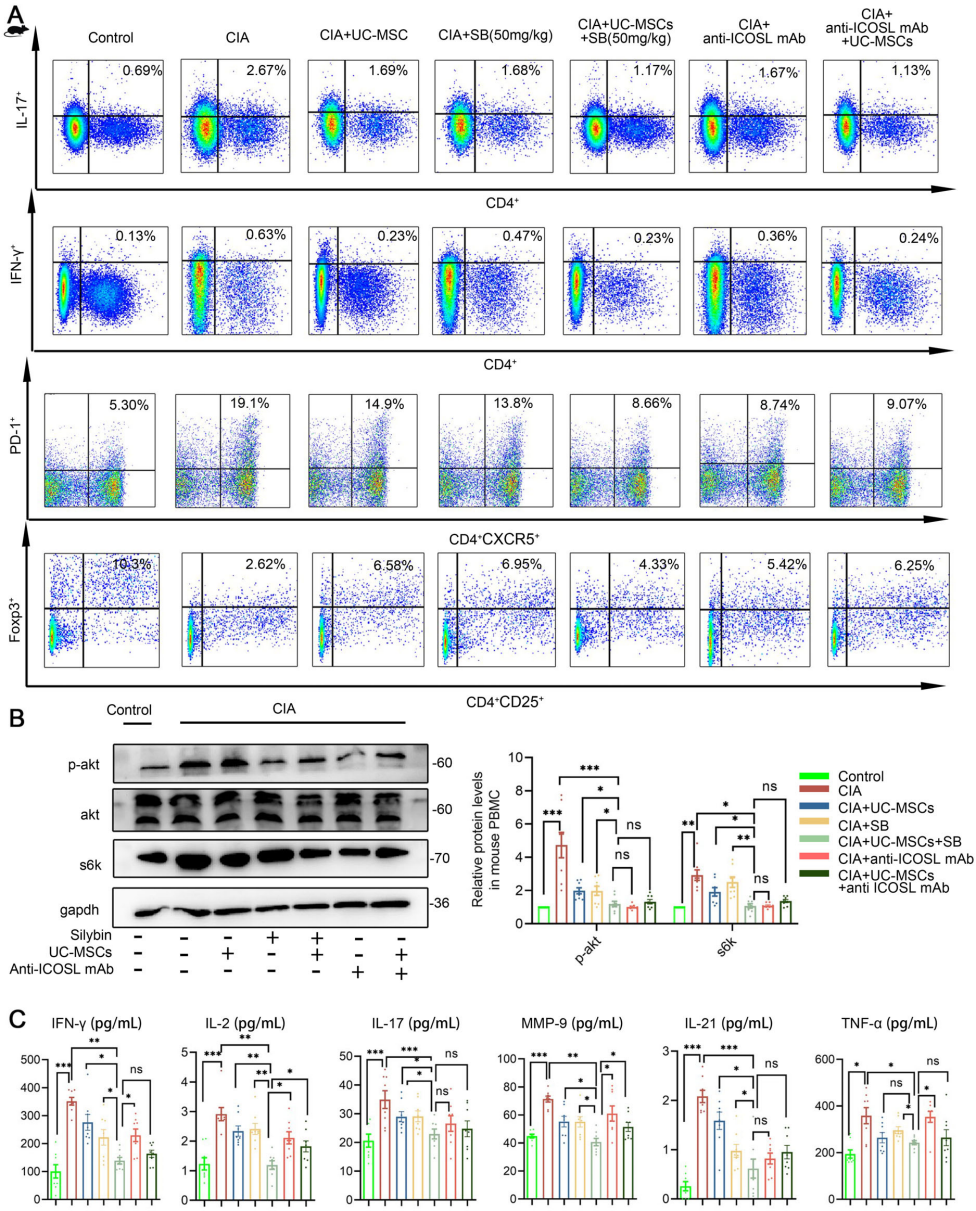
A combination strategy of UC‐MSCs and silybin alleviated arthritic symptoms in the CIA model via ICOS/ICOSL. (A) IL‐17A^+^CD4^+^Th17, IFN‐γ^+^CD4^+^Th1, and PD1^+^CXCR5^+^CD4^+^Tfh cells proportion of each group of in control and CIA mice splenocytes. (B) Western blot of p‐AKT, total AKT, and S6k in control and CIA mice‐PBMC. (C) Expression of inflammatory factors in each group of mice plasma. Data are represented as mean ± SEM (*n* = 8).

From an immunomodulatory perspective, we analyzed T cell subset in splenocytes and observed a significant reduction in Th17, Th1, and Tfh cell percentages in the combination group compared with the single‐treatment groups (Figure 7A). We further examined the protein levels of p‐AKT and S6K to validate the inhibitory impact on the PI3K/AKT/mTOR pathway and noted a corresponding decrease (Figure 7B). Additionally, serum cytokine levels, including IFN‐γ, IL‐1β, IL‐2, IL‐6, IL‐17, IL‐21, TNF‐α, and MMP9, were markedly reduced in the combination group, which also demonstrated enhanced cytokine suppression compared with individual treatments (Figure 7C). ICOSL blockade yielded consistent results in both in vivo and in vitro experiments. In summary, our data confirm that the combined approach of UC‐MSCs and SB offers superior therapeutic outcomes in collagen‐induced RA mouse models.

## 3 Discussion

UC‐MSCs have emerged as a promising therapeutic approach for RA due to their immunomodulatory and regenerative properties. Recent reviews have highlighted their potential in tissue engineering and their advantages over traditional RA treatments, such as NSAIDs, which fail to prevent joint destruction and often cause adverse effects [30, 31].

A comprehensive analysis of nonresponsive cases is crucial for refining therapeutic strategies and improving future assessments of treatment efficacy. Our study identified elevated ICOS expression in peripheral immune cells as a key barrier to UC‐MSC therapy responsiveness. This upregulation, in combination with ICOSL on UC‐MSCs, led to excessive T cell activation and the release of proinflammatory cytokines, potentially undermining the therapeutic benefits of UC‐MSCs. To address this challenge, we explored the combinatory use of SB and UC‐MSCs, which demonstrated enhanced immunoregulatory effects. These findings were validated both in vitro using patient‐derived samples and in vivo within the CIA model, highlighting a potential strategy to improve the efficacy of UC‐MSC therapy in RA.

Previous studies have highlighted the heterogeneity of UC‐MSCs, identifying distinct subpopulations with unique transcriptomic profiles and adaptive responses to changes in the tissue microenvironment. This inherent variability may contribute to the observed differences in therapeutic outcomes [32, 33]. Our study primarily focuses on the interaction between UC‐MSCs and the immune system, a key determinant of disease heterogeneity. Consistent with previous findings, our results confirm that the ICOS/ICOSL axis plays a pivotal role in initiating inflammatory pathways, shedding light on the underlying mechanisms of nonresponsiveness to UC‐MSC therapy in certain patients. To address this challenge, we explored a coadministration strategy aimed at disrupting ICOS/ICOSL‐mediated signaling. We systematically screened for compounds capable of binding to ICOS or ICOSL to prevent downstream inflammatory cascades and identified SB as a promising candidate. Notably, SB exhibited potential as a competitive binding inhibitor, effectively mitigating these detrimental effects. Moreover, its well‐documented anti‐inflammatory, antifibrotic, and lipid‐lowering properties have been shown to alleviate inflammation in arthritic models, further supporting its therapeutic potential in RA [34, 35]. However, similar to many traditional therapeutics, the precise mechanisms underlying SB's anti‐inflammatory effects in RA remain incompletely understood. Without a comprehensive elucidation of its mode of action, optimizing its clinical application remains challenging. In this study, we provide a scientific foundation for its therapeutic potential, offering mechanistic insights that may facilitate its rational integration into RA treatment strategies.

Interestingly, our results indicate that Treg cell numbers increase following UC‐MSC stimulation, which aligns with previous findings suggesting that ICOSL engagement may promote ICOS+ Treg expansion [36]. While our study primarily focuses on the detrimental effect of ICOS/ICOSL interaction in driving T cell activation and impairing UC‐MSC therapeutic efficacy, these findings suggest that additional mechanisms may be at play. It is possible that ICOS/ICOSL signaling exerts complex and context‐dependent effects on immune regulation, influencing both inflammatory and immunosuppressive pathways. Further investigation is required to delineate these mechanisms and to determine whether modulating ICOS/ICOSL signaling could enhance the therapeutic potential of UC‐MSCs in RA.

One limitation of this study is that we did not comprehensively assess potential drug interactions beyond the ICOS/ICOSL axis when integrating SB into the UC‐MSC treatment regimen. Additionally, SB's inherently low bioavailability poses a challenge to its therapeutic application. While higher doses may enhance its efficacy, they also increase the risk of adverse effects, including gastrointestinal disturbances and allergic reactions. Therefore, further rigorous investigations are required to determine an optimal balance between safety and efficacy, particularly in combination with MSC therapy. Moreover, given SB's lipid‐lowering properties, caution should be exercised in individuals receiving cholesterol‐lowering medications or those with inherently low cholesterol levels.

Medication responses vary significantly among individuals and often require personalized adjustments, which is a fundamental principle of clinical treatment. In this study, we present a comprehensive approach: first, identifying patients with limited responsiveness to MSC therapy; second, strategically selecting complementary pharmacological interventions to mitigate these limitations; and finally, optimizing therapeutic outcomes. This framework underscores both the necessity and feasibility of developing precision medicine strategies, paving the way for more targeted and effective treatments in the future.

## 4 Materials and Methods

### 4.1 RA Patients, HCs, and PBMCs Collection

The enrolled RA patients and HCs were all from the Affiliated Drum Tower Hospital of Nanjing University Medical School. A total of 26 RA patients were diagnosed based on the 2010 American College of Rheumatology diagnosis of RA. Patients were excluded if they had infections and tumors. Ten HCs from women of the same age with no abnormal blood examination, no autoimmune diseases, and no infection. Mononuclear cells from the peripheral blood of RA patients and HCs were isolated by Ficoll density gradient centrifugation (Lymphoprep, 07851; STEMCELL). Our study was approved by the Ethics Committee of the Affiliated Drum Tower Hospital of Nanjing University Medical School (No. 2021–544‐01). Participants gave their informed consent for all studies.

### 4.2 CIA Model and Treatment

Female DBA/1J mice (8‐week‐old) were purchased from Weitonglihua (Beijing, China) and housed in a specific pathogen‐free environment. Throughout the study, the animal experiments were divided into two parts.

In the first part, all mice were randomly divided into five groups of eight mice each: control, CIA, CIA+UC ‐MSC, CIA+sh ‐ICOSL UC ‐MSC, and CIA+UC‐MSC+PI3K inhibitor. On day 0, female DBA/1J mice were immunized by being injected subcutaneously at the base of the tail with bovine type II collagen (bCII) emulsified in complete Freund's adjuvant (CFA). On day 21, a booster injection of bCII/ incomplete Freund's adjuvant (IFA) was given to the mice. The UC‐MSCs of healthy people were from the Stem Cell Center of Jiangsu Province. UC‐MSCs at passages 5–7 were used for this research. UC‐MSCs or sh‐ICOSL UC ‐MSC injections were administered to mice of CIA+UC‐MSC, CIA+sh‐ICOSL UC‐MSC, and CIA+UC‐MSC+PI3K inhibitor group on day 18 and day 25 via tail vein at a dose of 5 × 10^5^ UC‐MSCs per mouse; and CIA+UC‐MSC+PI3K inhibitor group on day 18 and day 25 gavaged with PI3K inhibitor at a dose of 1 mg/kg per mouse.

In the second part, all mice were randomly divided into seven groups of eight mice each: control, CIA, CIA+UC‐MSC, CIA+SB (150 mg/kg), CIA+UC‐MSC+SB (150 mg/kg), CIA+anti‐ICOSL mAb (3 mg/kg), and CIA+anti‐ICOSL mAb (3 mg/kg)+UC‐MSCs. On day 0, female DBA/1J mice were immunized by being injected subcutaneously at the base of the tail with bCII emulsified in CFA. On day 21, a booster injection of bCII/IFA was given to the mice. The UC‐MSCs of healthy people were from the Stem Cell Center of Jiangsu Province. UC‐MSCs at passages 5–7 were used for this research. UC‐MSCs injections were administered to mice of CIA+UC‐MSC and CIA+UC‐MSC+SB (150 mg/kg) group on day 18 and day 25 via tail vein at a dose of 5 × 10^5^ UC‐MSCs per mouse. Anti‐ICOSL mAb (3 mg/kg) was injected twice a week in CIA+anti‐ICOSL mAb (3 mg/kg) and CIA+anti‐ICOSL mAb (3 mg/kg)+UC‐MSCs groups’ mouse. Figure S2A confirms the stimulatory effect of ICOSL on murine splenocytes. SB (C25H22O10, purity 98%) was purchased from Meryer Chemical Technology Co., Ltd (Shanghai, China). Polyethylene glycol 400 (PEG400) was purchased from Tokyo Chemical Industry Co., Ltd. (Tokyo, Japan). Cremophor EL (polyoxyethylene castor oil) was purchased from Aladdin Industrial Corporation (Shanghai, China). SB at dosages of 150 mg/kg was dissolved in the solution of 35% PEG400, 15% Cremophor EL, 5% ethanol, and 45% saline. Starting from day 1, mice in the CIA+SB (150 mg/kg) and CIA+UC‐MSC+SB (150 mg/kg) group were gavaged with SB solution at a dose of 150 mg/kg for 32 days. Figure S2B illustrates the antagonistic effect of SB on ICOSL‐induced p‐AKT activation, while Figure S2C displays the results of SB's cytotoxicity assay.

After the second immunization, mice were inspected daily, and disease was monitored by applying a clinical score to each paw (0 = normal; 1 = one toe inflamed and swollen; 2 = more than one toe, but not the entire paw inflamed and swollen or mild inflammation and swelling of entire paw; 3 = entire paw inflamed and swollen; and 4 = very inflammatory and swollen paw). Our operations conform to the Ethics Committee of the Affiliated Drum Tower Hospital of Nanjing University Medical School (No. 2020AE01061).

### 4.3 In Vitro Coculture System of UC‐MSCs, SB, and PBMCs

UC‐MSCs used in this study were counted and inoculated in the bottom of the well plate. After UC‐MSCs had adhered to the wall, SB with a dose of 100 µmol/L and a 10‐fold count of PBMCs was placed in the well plate. Twenty‐four hours later, the upper layer of suspended PBMCs and the medium were collected for subsequent experiments.

### 4.4 Construction of ICOS Knockdown, ICOS Overexpression, and ICOSL Knockdown Cell Lines

*Plasmid construction*: To construct a plasmid overexpressing human‐derived ICOS, we extracted the ICOS and ICOSL gene from human peripheral blood cDNA by PCR and cloned it into a third‐generation lentiviral shuttle vector by homologous recombination, and the plasmid was identified by sanger sequencing. To construct the shRNA plasmid for ICOS and ICOSL, we designed the target sequence for ICOS and ICOSL (Table S1), then ordered oligonucleotide primers from Kingsley, annealed them to form double‐stranded DNA with sticky ends, and cloned them into PLKO.1–TRC vector using T4 ligase. The efficacy of knockdown and overexpression is presented in Figure S3A.

*Lentiviral packaging*: HEK293T cells were transfected with PSPAX, PMD 2 g, and shuttle plasmid (pLKO.1 or pLenti) in a molar ratio of 1:1:1 for a total of 5 µg after growing at approximately 70–80% density in T25 culture bottles with Lipofectamine 3000 as transfection reagent. Viral supernatants were collected 48 h after transfection. After 48 h of transfection, the virus supernatant was collected, filtered through a 0.45 µM PVDF membrane, and subsequently infected with Jurkat T cells or UC‐MSCs. Jurkat T cells or UC‐MSCs were screened with the antibiotic puromycin for 1 week after 48 h of infection to obtain ICOS overexpressing or knockdown cell lines or ICOSL knockdown UC‐MSCs.

### 4.5 Real‐Time PCR

Cells were lysed with 1 mL TRIzol reagent and placed on ice for 5 min. After adding 200 µL chloroform, the lysate was vortexed and centrifuged at 12,000×*g* in 4°C for 10 min. The upper water phase was extracted and mixed with 500 µL isopropanol and centrifuged at 12,000×*g* in 4°C for 10 min. After that, the cDNA was diluted 10 times with DEPC water. The results were analyzed using the 2‐(△△Ct) method and the primer sequences used are shown in Tables S2 and S4.

### 4.6 Western Blot

Cells were collected and lysed by RIPA containing 1×protease inhibitor and 1×phosphatase inhibitor. The proteins were transferred onto 0.45 µm PVDF membrane and was blocked in 5% skim milk powder and placed on the shaker for 2 h. Antibody was placed on the shaker overnight at 4°C. The primary antibodies included anti‐beta actin antibody (ab8226; Abcam); anti‐S6K antibody (ab32529; Abcam); anti‐AKT antibody (ab941763; Abcam); anti‐AKT1 (phospho S473) antibody (ab81283; Abcam); anti‐ICOS antibody (ab224644; Abcam); anti‐ICOS ligand antibody (ab124972; Abcam). In the next day, the membrane was washed and then was incubated with the HRP‐conjugated antimouse or antirabbit IgG at room temperature for 1 h after the secondary antibody was diluted with 1×TBST according to the proportion provided in the manual. Subsequently, the membrane was washed and was visualized with chemiluminescent HRP substrate via chemo‐luminescence, and finally was further quantified using Image J software (NIH).

### 4.7 Elisa Assay

The levels of cell factor IL‐2, IL‐21, IFN‐γ, IL‐17, TNF‐α, and MMP‐9 in cell culture supernatants and mice serum were detected according to the manufacturer's instructions of ELISA kits.

### 4.8 Micro‐CT

On day 32, the hind legs of each group of mice were carefully removed from the skin and the legs were immersed in paraformaldehyde solution. The mice legs were subjected to micro‐CT scanning.

### 4.9 Flow Cytometry and Antibodies

Cell death dye and surface markers were first stained, and the cells were then fixed, permeabilized with an intracellular staining buffer set (Thermo Fisher Scientific) following the manufacturer's protocol, and stained with intracellular or intranuclear markers. Flow cytometry was performed using FACS Aria II (BD Biosciences), and the data were analyzed using Flow Jo v10.0.7 software (Tree Star Inc., Ashland, OR, USA). The antibodies used for flow cytometry are listed in Tables S1 and S3. The gating strategy for flow cytometry analysis is illustrated in Figures S3B–E and S4A–C.

### 4.10 SPR Experiment

The CM5 chip was installed according to the standard operating procedures of the Biacore 8k instrument prior to ligand immobilization. The target coupling volume can be calculated according to the following formula: *R*_max_ = analyte MW/ligand MW × RL × Sm, where *R*_max_ is the maximum binding capacity on the chip surface. For ligand pre‐enrichment, ligand proteins were diluted to 20 µg/mL with acetate buffers at pH 4.5, 5.0, and 5.5, and 100 µL of each solution was prepared for testing. The pH 5.5 was determined as the best coupling condition by the pre‐enrichment experiment. The concentration of the sample was determined by the results of the pre‐experiment, which was 200 µM diluted downward in six concentration gradients, namely, 200, 100, 50, 25, 12.5, and 6.25 µM. A 0 concentration was added. Samples were injected with a contact time of 120 s, a flow rate of 10 µL/min, and a dissociation time of 120 s. The data were analyzed using Biacore Insight Evaluation Software version 3.0.12.15655.

### 4.11 Molecular Modeling

To obtain the protein structure of ICOS, log on to the https://www.rcsb.org/ website, search for “ICOS,” download, and save it. To obtain the molecular structure of “silymarin,” log on to https://pubchem.nc‐bi.nlm.nih.gov/ website, download, and save it. The prediction of binding sites was done by using discovery studio software.

### 4.12 Histopathologic Analysis

On day 32, the hind legs of each group of mice were carefully removed from the skin and the legs were immersed in paraformaldehyde solution. The mice legs were subjected to HE staining and Safranin fast green staining for bone. HE staining results were as follows: nucleus was blue, and cytoplasm was red. Safranin fast green staining for bone results were as follows: cartilage was red or orange‐red, and bone formation was green; and some connective tissues were red.

### 4.13 Statistical Analysis

Statistical calculations were performed using GraphPad software or R. Statistical details for specific experiments including the tests used and value/definition are provided in figure legends. Box‐and‐whisker plots represent median, quartiles, and maximum/minimum values. Bar charts and error bars indicate means and SEM. In vitro assays were performed with 3–4 technical replicates per condition. Sample sizes for in vivo mouse studies were based on the number of mice routinely needed to establish statistical significance based on variability within study arms. Each data point represents either a technical replicate (in the case of in vitro studies) or a biological replicate (individual mouse from in vivo studies). *p* Values and FDR values <0.05 were considered in all analyses to be statistically significant.

## Author Contributions

Lingyun Sun and Yizhun Zhu conceived and raised funds for the study. Xin Wen, Yuchun Wang, Ding Shuai, and Yafeng Wang supervised the overall project. Han Xie collected clinical samples and information. Yuchun Wang carried out animal experiments with advice of Shanshan Liu. Hongwei Chen and Yuchun Wang performed cell biological experiments with the advice of Dan Wu and Ying Xie. Yuchun Wang and Shuai Ding integrated the data and wrote the drafts of the manuscript. All authors edited the manuscript. All authors have read and approved the final manuscript.

## Conflicts of Interest

Author Yizhun Zhu is an Editorial Board member of MedComm. Author Yizhun Zhu was not involved in the journal's review of or decisions related to this manuscript. The other authors declare no conflicts of interest.

## Ethics Statement

This study was approved by the Ethics Committee of Affiliated DrumTower Hospital and experimented with its guidelines. Human experiments are carried out under the project license (No. 2021‐544‐01), and animal experiments are carried out under the project license (No.2020AE01061). And informed consent was obtained from all subjects.

## Supporting information



Supporting Information

## Data Availability

All data used in this study can be acquired by contacting the corresponding authors.
