# Supplementary material for: A Potential Approach of Mesenchymal Stem Cells Combined Silybin for Synergistic Treatment in Rheumatoid Arthritis via ICOS/ICOSL
Source: MedComm (2020). 2025 Oct 28;6(11):e70450. doi: 10.1002/mco2.70450 (PMC12559906; doi:10.1002/mco2.70450)
Supplement: Supplementary file 1 — Supporting Information [file MCO2-6-e70450-s001.pdf]

A Potential Approach of Mesenchymal Stem Cells Combined Silybin For Synergistic Treatment in Rheumatoid Arthritis Via ICOS/ICOSL

Yu Chun Wang1,2,3, #, *, Shuai Ding3, #, Ya Feng Wang3, #, Han Xie2,3, #, Gen Hong Yao3, Shan Shan Liu3, Hong Wei Chen3, Dan Wu3, Ying Xie4, Xin Wen3, *, Yi Zhun Zhu2, *, Ling Yun Sun2,3, *

1 The Key Laboratory of Biochemistry and Molecular Pharmacology, Chongqing Medical University, Chongqing, China, 400010

2 School of Pharmacy, Faculty of Medicine, Macau University of Science and Technology, Macau SAR, China, 999078

3 Department of Rheumatology and Immunology, Nanjing Drum Tower Hospital, Macau University of Science and Technology, Macau SAR, China, 999078

4 The Second Affiliated Hospital of Guangzhou University of Chinese Medicine, Guangzhou, Guangdong, China, 510000

*Correspondence

#These authors have contributed equally to this work and share the first authorship.

Supply Figures

Figure S1

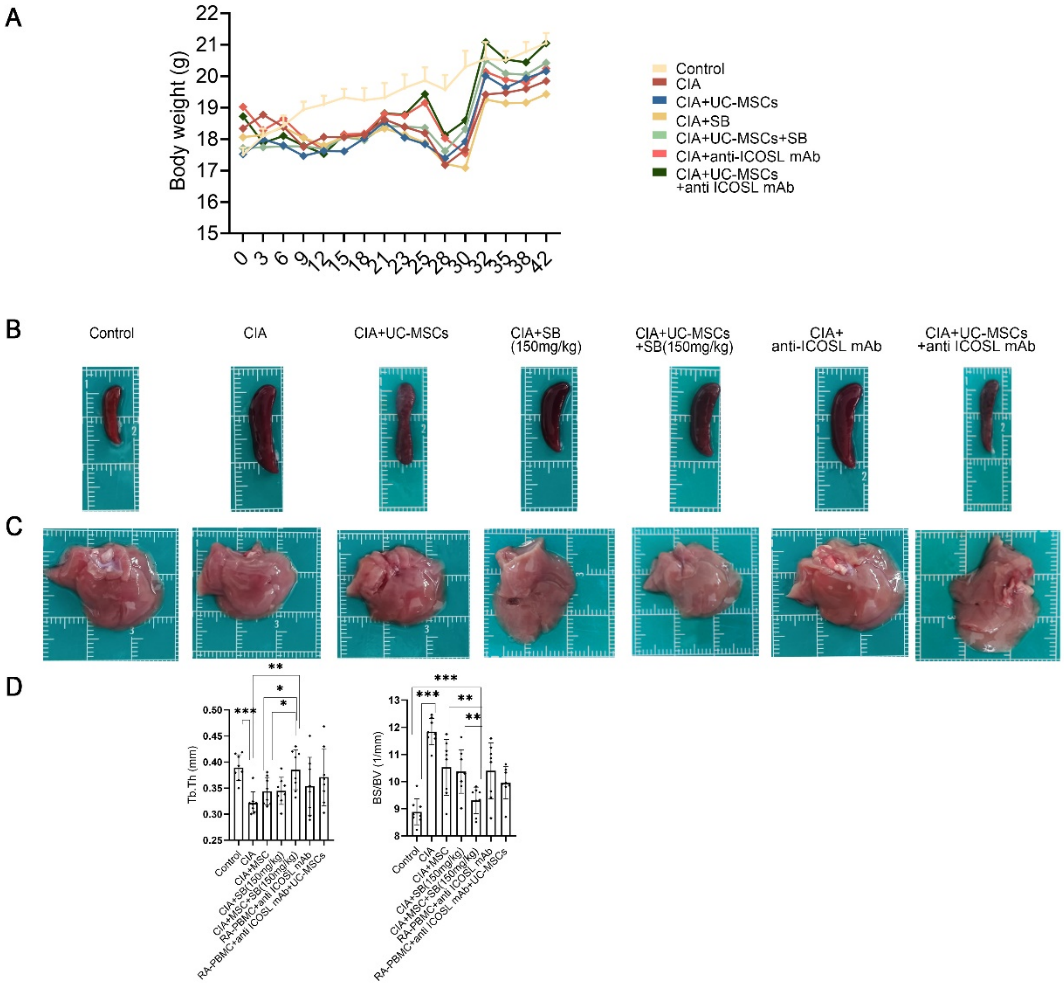

(A) Body weight changes across different groups.

(B) Spleen morphology in each group.

(C) Liver morphology in each group.

(D) Statistical analysis of skeletal parameters among groups.

Figure S2

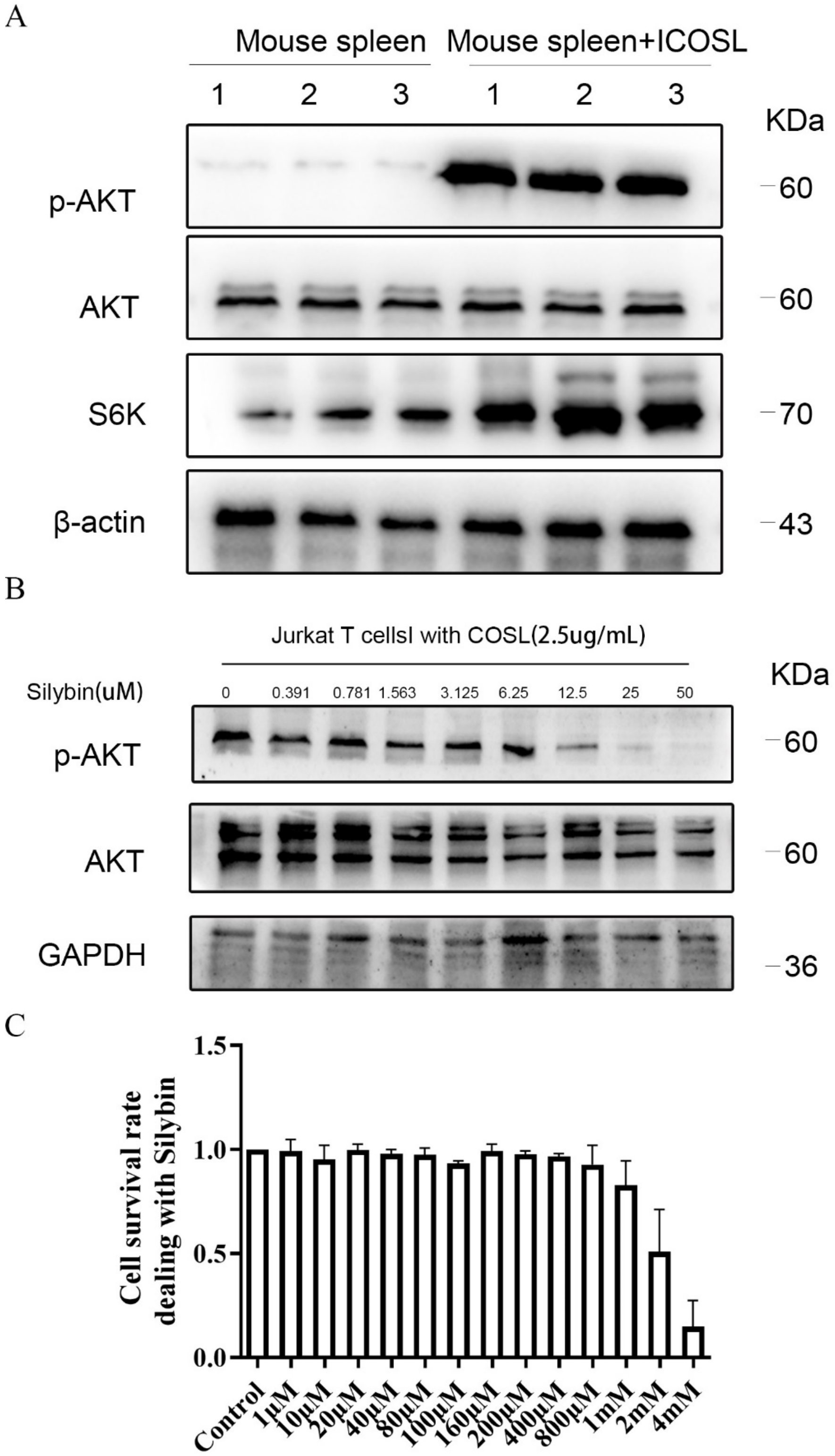

(A) Mouse spleen cells were stimulated with ICOSL (2.5 μg/mL) to verify that human ICOSL was also reacted with mice

(B) Western blot showing the dose-dependent inhibitory effect of Silybin on p-AKT expression.

(C) Cytotoxicity assay evaluating the viability of cells treated with different concentrations of Silybin to define its safe dosage range.

Figure S3

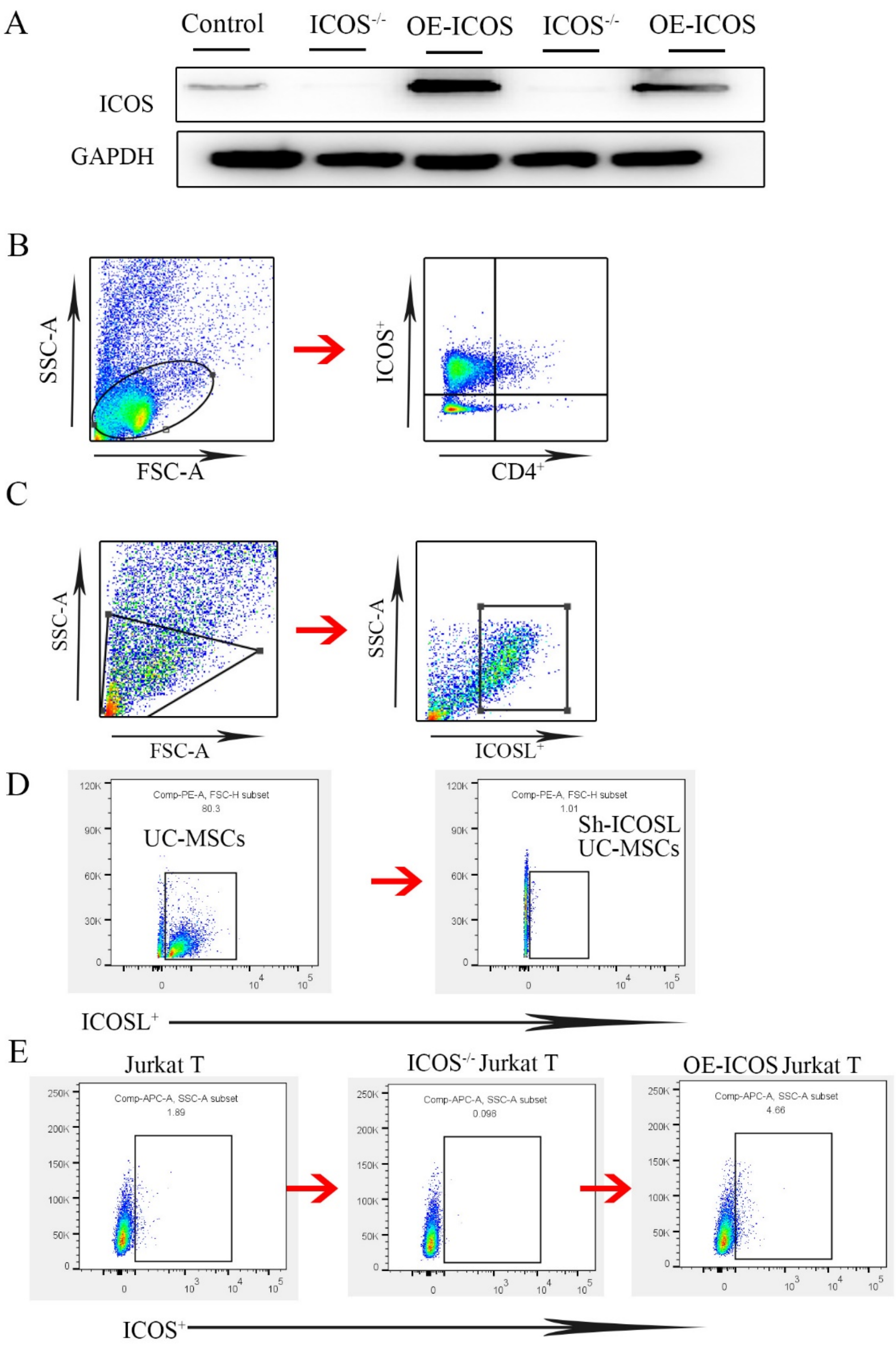

(A) Western blot confirming ICOS expression levels in Jurkat T cells following ICOS knockdown (ICOS-/-) and overexpression (OE-ICOS).

(B) Flow cytometry gating process of $ICOS^+CD4^+$ cells in RA PBMC

(C) Flow cytometry gating process of $ICOSL^+$UC-MSCs

(D) The knockdown efficiency of ICOSL on UC-MSCs was greater than 90%

(E) The knockdown efficiency of ICOS on Jurkat T was greater than 90%, and the overexpression efficiency was about 200%

Figure S4

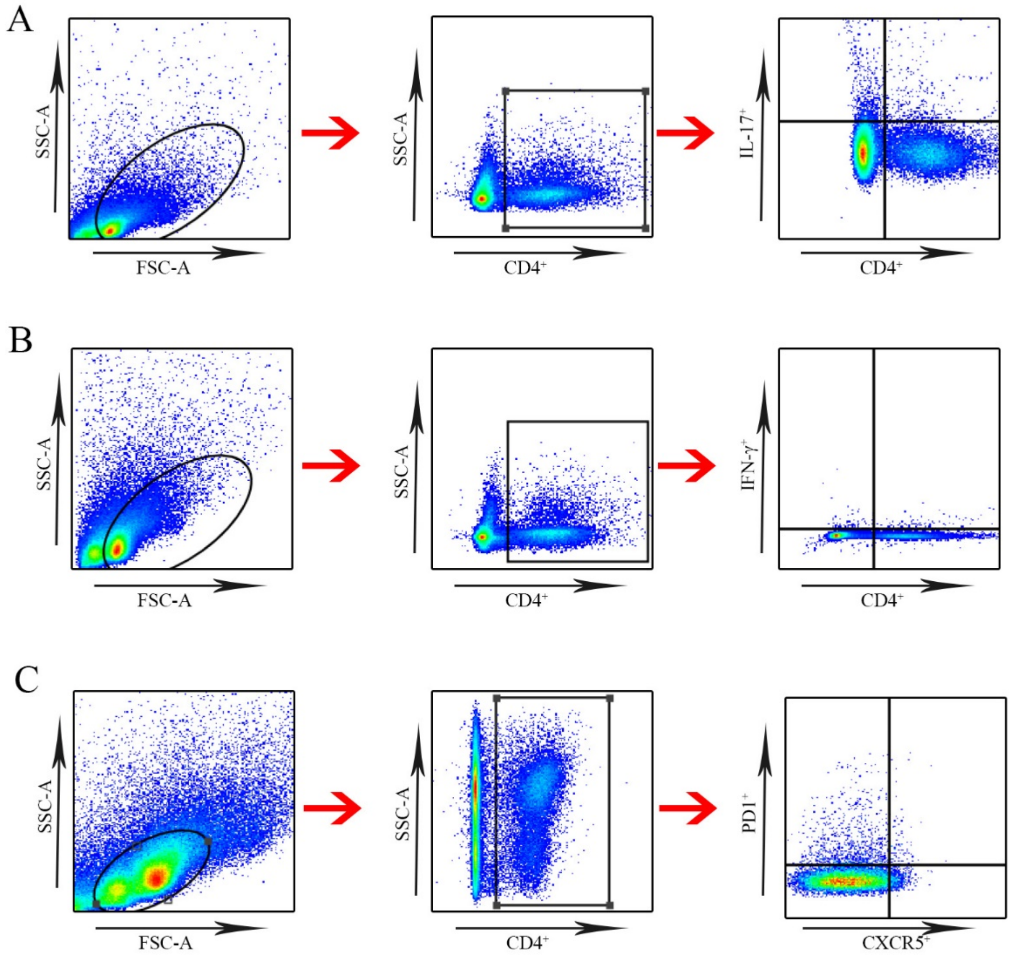

(ABC) Flow cytometry gating process of Th17/Th1/Tfh in control and CIA mice spleen

Supplemental tables

Table S1: Target sequence for ICOS

Gene	Primer Sequence(5'to3')
shhICOS-1	GCATACTTATTTGTTGGCTTA
shhICOS-2	CCTTTGTTGTAGTCTGCATTT
shhICOS-3	CTGCCAATTATGAGATGTTTA
shhICOS-4	CCATTCTCATGCCAACTATTA
shhICOS-5	GCTGAAGTTCTGGTTACCCAT
shhICOSL	CCCAACGTGTACTGGATCAAT

Table S2: Primers used for RT-qPCR validation

Gene	Primer Sequence(5'to3')
Human ICOS	Forward: CCCATAGGATGTGCAGCCTTTG
	Reverse: GGCTGTGTTCACTGCTCTCATG
Human GAPDH	Forward: ATGGGGAAGGTGAAGGTCG
	Reverse: GGGGTCATTGATGGCAACAATA

Table S3: Antibodies Used in the Study

Antibody	Catalog No.	Supplier
eBioscience™ Fixable Viability Dye eFluor™ 506	65-0866-14	Invitrogen™
PerCP/Cyanine5.5 anti-human CD4 Antibody	317427	BioLegend
Pacific Blue™ anti-human CD185 (CXCR5) Antibody	356917	BioLegend
FITC anti-human CD279 (PD-1)	329904	BioLegend
APC anti-human/mouse/rat CD278 (ICOS)	313510	BioLegend
Recombinant Anti-ICOS antibody	ab224644	Abcam
Recombinant Anti-beta Actin antibody	ab8226	Abcam
PE anti-human CD275 (B7-H2, ICOSL)	506925	BioLegend
Recombinant Anti-ICOS Ligand antibody	ab124972	Abcam
Recombinant Anti-S6K antibody	ab32529	Abcam
Recombinant Anti-AKT1 (phospho S473) antibody	ab81283	Abcam
APC anti-human CD4 Antibody	300514	BioLegend
FITC anti-human CD4 Antibody	300506	BioLegend
APC anti-human CD185 (CXCR5) Antibody	356907	BioLegend
PE anti-human CD279 (PD-1) Antibody	329905	BioLegend
FITC anti-human IFN-γ Antibody	502506	BioLegend
PE anti-human IL-17A Antibody	512306	BioLegend

Antibody	Catalog No.	Supplier
FITC anti-mouse CD4 Antibody	100406	BioLegend
PE anti-mouse IL-17A Antibody	506903	BioLegend
PerCP/Cyanine5.5 anti-mouse IFN-γ Antibody	505822	BioLegend
PerCP/Cyanine5.5 anti-mouse CD4 Antibody	100433	BioLegend
APC anti-mouse CD185 (CXCR5) Antibody	145505	BioLegend
PE anti-mouse CD279 (PD-1) Antibody	109103	BioLegend

Table S4: Primer Sequences Used in This Study

Gene (Target)	Forward Primer (5'-3')	Reverse Primer (5'-3')
h-CD27	ACTACTGGGCTCAGGGAAAGCT	GGATCACACTGAGCAGCCTTTC
h-CD70	TTCGCACAGGCTCAGCAGCAG	TTGTCCAGCTCTGGTCCATGCA
h-CD28	GAGAAGAGCAATGGAACCATTATC	TAGCAAGCCAGGACTCCACCAA
h-CD80	CTCTTGGTGCTGGCTGGTCTTT	GCCAGTAGATGCGAGTTTGTGC
h-CD86	CCATCAGCTTGTCTGTTTCATTCC	GCTGTAATCCAAGGAATGTGGTC
h-ICOS	CCCATAGGATGTGCAGCCTTTG	GGCTGTGTTCACTGCTCTCATG
h-ICOSL	GTTTCACTGCCTGGTGTTGAGC	ACGACGGGCACGCTGAAGTTTG
h-CD137	TCTTCCTCACGCTCCGTTTCTC	TGGAAATCGGCAGCTACAGCCA
h-CD137L	GCCTCTTGGACCTGCGGCAG	CGTGTCCTCTTTGTAGCTCAGG
h-OX-40	CCTACATCTGCCTGCACTTCTC	TGATGACTGAGTTGTTCTGCACC
h-OX-40L	CCCAAGCCTATGTGCGTGGGGGCTCGGCG	CGGAATTCTCAGATCTTGGCCAGGGTG
human-CD40	CCTGTTTGCCATCCTCTTGGTG	AGCAGTGTTGGAGCCAGGAAGA
human-CD40L	GCGGCACATGTCATAAGTGAGG	GTCCTTGTCTTTTAACGGTCAGC
human-PD1	AAGGCGCAGATCAAAGAGAGCC	CAACCACCAGGGTTTGGAACTG
human-PDL1	TGCCGACTACAAGCGAATTACTG	CTGCTTGTCCAGATGACTTCGG
human-TIM-3	GACTCTAGCAGACAGTGGGATC	GGTGGTAAGCATCCTTGGAAAGG
human-Lag-3	GCAGTGTACTTCACAGAGCTGTC	AAGCCAAAGGCTCCAGTCACCA
human-TNFRSF14	TTCTCTCAGGGAGCCTCGTCAT	CTCACCTTCTGCCTCCTGTCTT
human-CD96	TGTATGGCTCTGTCTCCAGTCC	CTGGAGAAGGTTGGGTGTCAAG
human-CD100	AGCTCTGCACAAAGCCATCAGC	CCAGCATAGACAAACCTGTTGCC

Gene (Target)	Forward Primer (5'-3')	Reverse Primer (5'-3')
human-CD226	GGTGATACAGGTGGTTCAGTCAG	GGCTGGATCTTTTCCCACCTCA
human-GITR	CCAGTGTATCGACTGTGCCTCG	CACAGCGTTGTGGGTCTTGTTC
human-TNFSF14	GGTCTCTTGCTGTTGCTGATGG	TTGACCTCGTGAGACCTTCGCT
human-CD276	CTGGCTTTCGTGTGCTGGAGAA	GCTGTCAGAGTGTTTCAGAGGC
human-B7-H4	CTCACAGATGCTGGCACCTACA	GCAAGGTCTCTGAGCTGGCATT
human-CD28H	CAAAGGGACTCAGGTAACAGCC	GGAGGTTGAATAAATGCTCTGGC
human-CEACAM1	CACGCCAATAACTCAGTCACTGG	TTGTGGAGCAGGTCAGGTTCAC
human-HHLA2	GGATTTTGGTGCCCTCTGCGAT	TCTTGTTGGGCTCCATCAGCAG
